# Prevalence of vertebral fractures at death

**DOI:** 10.1007/s00774-025-01577-z

**Published:** 2025-01-13

**Authors:** Noriko Ogawa, Masahiro Yamamoto, Rie Kobayashi, Atsuko Kawamura, Akihiro Matsumoto, Hiroki Otani, Keizo Kanasaki

**Affiliations:** 1 Department of Internal Medicine 1, Shimane University Faculty of Medicine, Shimane, 89-1 Enya-cho, Izumo, Shimane 693-8501 Japan; 2 Department of Developmental Biology, Shimane University Faculty of Medicine, Shimane, 89-1 Enya-cho, Izumo, Shimane 693-8501 Japan; 3 The University of Shimane Faculty of Nursing and Nutrition, 151 Nishihayashigi-cho, Izumo, Shimane 693-8550 Japan

**Keywords:** Vertebral fracture, Hip fracture, Autopsy imaging computed tomography, Osteoporosis

## Abstract

**Introduction:**

Despite many studies on the prevalence of vertebral fractures (VFs), the VF prevalence at death in the Japanese population remains unclear.

**Materials and methods:**

We evaluated the VF prevalence at death in a Japanese cohort using autopsy imaging computed tomography (AiCT). We enrolled 365 cadavers (188 men, 177 women, mean age of 84.6 years) donated for anatomical dissection at Shimane University School of Medicine. The VFs were diagnosed using the semiquantitative technique of Genant from the first cervical vertebra to the fifth lumbar vertebra.

**Results:**

The overall VF prevalence was 69.6% (58.5%/81.4% in men/women), of which 46.0% (29.8%/63.3% in men/women) had thoracic VFs, and 58.1% (50.5%/66.1% in men/women) had lumbar VFs. The most frequent fracture site was lumbar spine 1 (L1) with 31.5% (22.9%/40.7% in men/women), followed by thoracic spine 12 (T12) with 31.0% (20.7%/41.8% in men/women). In terms of severity, 3.8% (4.8%/2.8% in men/women), 23.8% (27.1%/20.3% in men/women), and 41.9% (26.6%/58.2% in men/women) were Grades 1, 2, and 3. The VFs from T3 to L5 and of Grade 3 severity were significantly higher in women. VF and Grade 3 fractures were associated with a history of surgical intervention for femoral neck fractures. VFs were not associated with the following underlying causes of death: cancer, heart disease, senile death, cerebrovascular disease, pneumonia, and aspiration pneumonia.

**Conclusion:**

The VF prevalence at death, assessed by AiCT in cadavers donated for anatomical dissection, was higher in both men and women compared with previous studies conducted on individuals aged ≥ 80 years in Japan.

## Introduction

The average life expectancy in Japan has been increasing annually, and it has been lower than the previous year in the past 2 years due to the COVID-19 pandemic. The Ministry of Health, Labour, and Welfare in Japan has reported that the life expectancy in 2019 was 81.41 and 87.45 years for men and women, respectively [1]. Although the life expectancy has increased, healthy life expectancy, defined as the average number of years a person can be expected to live in good health, was 72.68 and 75.38 years for men and women in 2019, respectively [2]. This represents a discrepancy of 8.73 and 12.06 years for men and women, respectively, frequently necessitating long-term care. The primary cause of necessity for long-term care is musculoskeletal diseases, including osteoporosis, accounting for approximately one-fifth of the total. Therefore, osteoporosis should be further studied and prevented to increase healthy life expectancy. To assess the current status of osteoporosis, large-scale community cohorts have been or are being conducted. However, these observational studies face limitations, such as loss to follow-up and the exclusion of individuals who cannot participate due to osteoporosis. Since the prevalence of osteoporosis increases with age, these studies tend to underestimate its occurrence among older populations. Moreover, vertebral fractures (VFs) in these studies are typically diagnosed using radiography. Computed tomography (CT) sagittal imaging provides a more accurate assessment of VFs compared to standard radiography [3], which may lead to underestimation of VF prevalence in these cohorts.

In this study, VFs were assessed using autopsy imaging computed tomography (AiCT) on cadavers donated for anatomical dissection at the Shimane University Faculty of Medicine. Whole-body CT scans were performed without concern for radiation exposure. All thoracic and lumbar VFs were assessed to determine the lifetime prevalence of VFs in this Japanese cohort.

## Materials and methods

### Participants

The AiCT center at Shimane University Faculty of Medicine was inaugurated in June 2011 for taking CT images of cadavers donated for gross anatomy dissection and those who have died at Shimane University Hospital for pathological clarification. Our study subjects were 365 donated cadavers (188 men and 177 women) from July 1, 2011, to December 31, 2019. Most participants had lived in Shimane Prefecture. In Japan, cadaver donation involves members of the public voluntarily donating their bodies without compensation to support the education and training of medical and dental students. Individuals who wish to donate register with a charitable association for cadaver donation at a local medical or dental school during their lifetime. The participants in this study were members of the charitable association at our university, primarily residents of Shimane Prefecture, residing in various regions within the prefecture.

### Assessment of VFs

VFs were evaluated using the internationally recognized Genant semiquantitative method [4]. Vertebral height was measured, and a reduction of 20–25% was determined to indicate Grade 1: mild fracture, a 25–40% reduction as Grade 2: moderate fracture, and a reduction of > 40% as Grade 3: severe fracture. Two observers, including a board-certified endocrinologist, evaluated the presence and severity of fractures. Disagreements were resolved by repeating measurements and reaching a consensus between the two observers.

### Statistical analysis

All data were presented as the mean ± standard deviation for each index. Mann–Whitney *U* test was used to statistically assess the age and number of VFs in both groups. Chi-squared test was used to assess the other remaining indices. Statistical analyses were conducted using IBM SPSS Statistics version 28.0.0.0, and the threshold for statistical significance was set at *P* < 0.05. Statistical significance was considered at *P* < 0.05.

### Ethics approval

The Medical Research Ethics Committee, Shimane University Faculty of Medicine approved this study (No. 20201008-1). Because the study made use solely existing information for medical education within the Department of Anatomy, Shimane University Faculty of Medicine and did not utilize samples obtained from the human body, the procedure for obtaining informed consent from the research subjects was not required following the provisions by the Ethical Guidelines for Medical and Health Research Involving Human Subjects.

## Results

### Patient characteristics

Table 1 shows the characteristics of the participants. The mean age at death was 84.6 years, with a mean of 82.2 and 87.1 years in men and women, respectively. Approximately 70% of participants died in hospitals, 26% in facilities, and 7% at home. The underlying cause of death was cancer, followed by senile death, heart disease, pneumonia, aspiration pneumonia, and cerebrovascular disease. Hypertension was the most frequent comorbidity. The mean age at death was significantly higher for women. The place of death was significantly more common in hospitals and facilities in men and women, respectively. The incidence of senile death was significantly higher in women, whereas death from pneumonia was more prevalent in men. Diabetes mellitus and chronic obstructive pulmonary disease (COPD) were significantly more prevalent in men.

**Table 1:** Characteristics of participants

	Total	Men	Women	*P* value
Number of subjects	365	188	177	
Age (years)	84.6 ± 10.3	82.2 ± 9.5	87.1 ± 10.5	< 0.001***^ǂ^
Place of death (%)				
Hospital	247 (67.7)	139 (73.9)	108 (61.0)	0.008**
Home	25 (6.9)	17 (9.0)	8 (4.5)	0.087
Facility	93 (25.5)	32 (17.0)	61 (34.5)	< 0.001***
Underlying Cause of death (%)				
Cancer	75 (20.6)	39 (20.7)	36 (20.3)	0.924
Heart disease	58 (15.9)	32 (17.0)	26 (14.7)	0.554
Senile death	68 (18.6)	17 (9.0)	51 (28.8)	< 0.001***
Cerebrovascular disease	13 (3.6)	4 (2.1)	9 (5.1)	0.128
Pneumonia	42 (11.5)	29 (15.4)	13 (7.3)	0.016*
Aspiration pneumonia	18 (4.9)	10 (5.3)	8 (4.5)	0.724
Comorbidity (%)				
Hypertension	130 (35.6)	62 (33.0)	68 (38.4)	0.279
Cerebrovascular disease	98 (26.9)	44 (23.4)	54 (30.5)	0.126
Diabetes mellitus	56 (15.3)	42 (22.3)	14 (7.9)	< 0.001***
COPD	45 (12.3)	30 (16.0)	15 (8.5)	0.030*
Chronic kidney disease	44 (12.1)	25 (13.3)	19 (10.7)	0.453
Coronary artery disease	60 (16.4)	33 (17.6)	27 (15.3)	0.554
Hyperlipidemia	26 (7.1)	13 (6.9)	13 (7.3)	0.873
Autoimmune disease	24 (6.6)	13 (6.9)	11 (6.2)	0.788
Thyroid disease	12 (3.3)	4 (2.1)	8 (4.5)	0.200

^ǂ^Mann–Whitney *U* test, others were analyzed using chi-square test, *COPD* chronic obstructive pulmonary disease

*P < 0.05, **P < 0.01, ***P < 0.001

### Number and severity of VFs

Table 2 shows the VF prevalence, which is 69.6% overall, with 58.5% and 81.4% in men and women, respectively. Of these, 46.0% and 58.1% had thoracic and lumbar VFs, respectively. The mean number of VFs was 2.5 overall, with 1.5 and 3.4 in men and women, respectively. Women had a significantly higher prevalence and number of VFs. The severity was classified as Grades 1, 2, and 3 in 3.8%, 23.8%, and 41.9%, respectively. The incidence of Grade 3 severe fractures was significantly higher in women. Previous osteosynthesis, including the use of plate, screw, and intramedullary nailings, and bipolar hip arthroplasty, was defined as a history of surgical intervention for hip fractures. The surge history of hip fractures was found in 14.3% of participants, which was significantly higher in women. The presence of lumbar spine 6 (L6) was 5.2% overall, with a higher incidence in males.

**Table 2:** Number and severity of VFs

	Total	Men	Women	*P* value
Prevalence of VFs (%)	254 (69.6)	110 (58.5)	144 (81.4)	< 0.001***
Number of VFs	2.45 ± 2.99	1.53 ± 2.22	3.42 ± 3.37	< 0.001***^ǂ^
Prevalence of thoracic VFs	168 (46.0)	56 (29.8)	112 (63.3)	< 0.001***
Prevalence of lumbar VFs	212 (58.1)	95 (50.5)	117 (66.1)	0.003**
Grade 1	14 (3.8)	9 (4.8)	5 (2.8)	0.329
Grade 2	87 (23.8)	51 (27.1)	36 (20.3)	0.128
Grade 3	153 (41.9)	50 (26.6)	103 (58.2)	< 0.001***
HSI for femoral neck fractures	52 (14.3)	14 (7.5)	38 (21.5)	< 0.001***
Exist of L6	19 (5.2)	14 (7.5)	5 (2.8)	0.047*

*VFs* vertebral fractures, *HSI* a history of surgical intervention, *L* lumbar spine

^ǂ^Mann–Whitney *U* test, others were analyzed using chi-square test

*P < 0.05, **P < 0.01, ***P < 0.001

### Site and severity of VFs

**Fig. 1:**
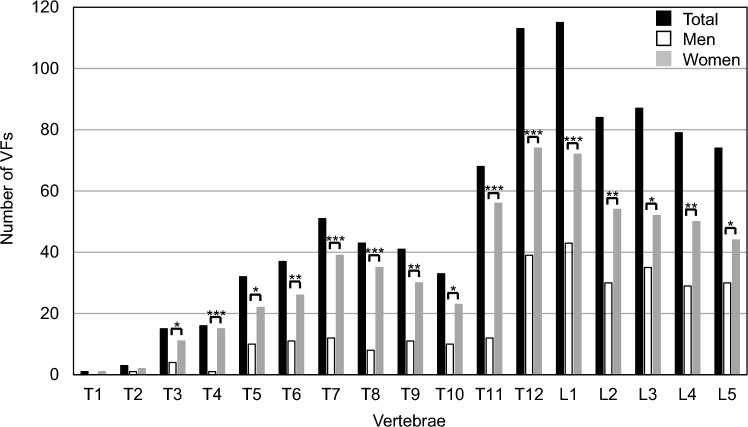
Prevalence of VF by site. Black squares represent the total, white squares for men, and gray squares for women. *P < 0.05, **P < 0.01, ***P < 0.001

**Fig. 2:**
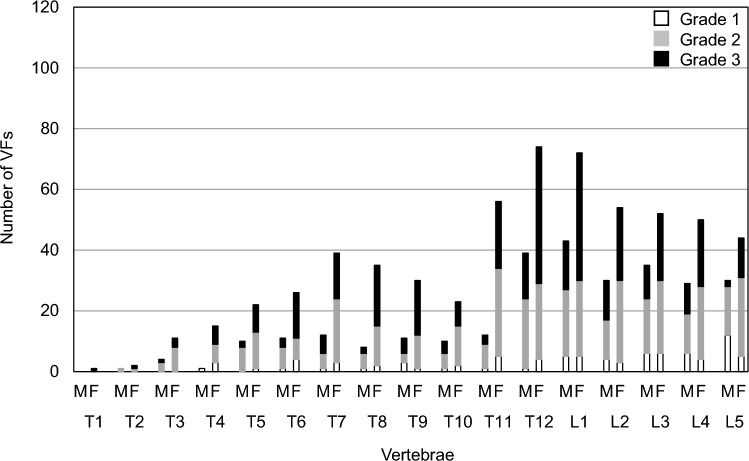
Severity of VFs by site. White squares represent Grade 1, gray squares for Grade 2, and black squares for Grade *M* male, *F* female

Table 3 and Fig. 1 show VF prevalence by site. The VF prevalence based on site showed a bimodal distribution at the thoracic–lumbar junction and in the middle thoracic lesions. The incidence of L1 fracture was the highest, followed by that of thoracic spine 12 (T12). A fracture at T7 was observed subsequent to that at T11 and T12. Moreover, the occurrence of VFs from T3 to L5 was significantly higher in women. Figure 2 shows the severity of VFs based on the affected site. Women showed a higher severity at all vertebral levels.

**Table 3:** Prevalence of VFs according to the site

Site of VFs	Total	Men	Women	*P* value
T1 (%)	1 (0.3)	0 (0.0)	1 (0.6)	0.302
T2	3 (0.8)	1 (0.5)	2 (1.1)	0.527
T3	15 (4.1)	4 (2.1)	11 (6.2)	0.049*
T4	16 (4.4)	1 (0.5)	15 (8.5)	< 0.001***
T5	32 (8.8)	10 (5.3)	22 (12.4)	0.016*
T6	37 (10.1)	11 (5.9)	26 (14.7)	0.005**
T7	51 (14.0)	12 (6.4)	39 (22.0)	< 0.001***
T8	43 (11.8)	8 (4.3)	35 (19.8)	< 0.001***
T9	41 (11.2)	11 (5.9)	30 (17.0)	< 0.001***
T10	33 (9.0)	10 (5.3)	23 (13.0)	0.011*
T11	68 (18.6)	12 (6.4)	56 (31.6)	< 0.001***
T12	113 (31.0)	39 (20.7)	74 (41.8)	< 0.001***
L1	115 (31.5)	43 (22.9)	72 (40.7)	< 0.001***
L2	84 (23.0)	30 (16.0)	54 (30.5)	0.001**
L3	87 (23.8)	35 (18.6)	52 (29.4)	0.016*
L4	79 (21.6)	29 (15.4)	50 (28.3)	0.003**
L5	74 (20.3)	30 (16.0)	44 (24.9)	0.035*

*T* thoracic spine

*P < 0.05, **P < 0.01, ***P < 0.001

### Association between VFs and a history of surgical intervention for hip fractures

Table 4 shows the correlation between VFs and history of surgical intervention for hip fractures. A surgical history of hip fractures was associated with an increased number and severity of thoracic and lumbar VFs. VFs and underlying causes of death were not significantly associated (cancer, heart disease, senile death, cerebrovascular disease, pneumonia, and aspiration pneumonia) (data was not shown).

**Table 4:** Association between VFs and a history of surgical intervention for femoral neck fractures

	Femoral neck fractures (–) *N* = 314	Femoral neck fractures (+) *N* = 51	*P* value
VFs (%)	207 (65.9)	47 (92.2)	< 0.001***
Thoracic VFs	129 (41.1)	39 (76.5)	< 0.001***
Lumbar VFs	168 (53.5)	44 (86.3)	< 0.001***
Grade 1	14 (4.5)	0 (0.0)	0.124
Grade 2	79 (25.2)	8 (15.7)	0.141
Grade 3	114 (36.3)	39 (76.5)	< 0.001***

*P < 0.05, **P < 0.01, ***P < 0.001

## Discussion

In the present study, we revealed for the first time the VF prevalence of all thoracic and lumbar vertebrae at death using AiCT of cadavers donated for anatomical dissection. This prevalence was higher than that reported in previous studies conducted in the same age groups.

VF prevalence in Japan has been documented in population-based cohort studies using standard radiographs. The VF prevalence was 30–40% in Japanese women in their 70 s in studies conducted in 1995 and 2001 [5–7]. The ROAD study, a large-scale residential cohort investigation, is currently underway in Japan, aiming to elucidate an epidemiology of locomotor disorders. The third survey in 2019 reported that the prevalence increased with age. Specifically, 41.5% of men and 53.0% of women aged ≥ 80 years had VFs [8]. Furthermore, a residential cohort in Miyagawa Village (Odaicho), Mie prefecture, which has been under investigation since 1997 demonstrated that the VF prevalence was 31% in their 80 s in the 2019 survey [9]. In this study, approximately 60% of men and 80% of women among residents with an average age in their 80 s at death had VFs, with a higher prevalence in both sexes compared with the previous reports about the VF prevalence in the 80 s [8, 9]. VF prevalence has been decreasing over the past two decades due to improvements in nutrition, a reduction in smoking rates, improved physical condition, and the advent of effective osteoporosis medications [9]. Our study participants include deaths from the previous approximately 10 years. The VF prevalence in this study was higher than that reported by Yamada et al. in 2009 or 2011, about 10 years ago, which was 54% in their 80 s.

The VF prevalence in previous cohort studies may have been underestimated due to the surveys being conducted with research subjects who were competent to participate in the survey, and the use of standard radiographs for assessment. In this study, the presence of VFs was assessed at death. Consequently, the lifetime prevalence of VFs could be elucidated, including the period when the subjects required care and could not participate in the cohort study. Additionally, we believe that using AiCT sagittal images provided a more accurate diagnosis of VFs than standard radiographs. Recently, sagittal CT images have been used to assess VFs internationally [10, 11], and efforts have been made to enhance VF diagnosis using artificial intelligence on CT images [12, 13]. While radiation exposure from CT scans is higher than that of radiographs, CT is increasingly being used in medical examinations, and its use in detecting VFs could improve future prevalence studies and early VF diagnosis.

In the present study, we demonstrated a significantly higher prevalence of both the number and severity of VFs in women. Severe VFs were more prevalent in women, which is consistent with the findings of the ROAD study [8]. Prevalence and severity of VFs in this study may have been affected by declining estrogen levels [14, 15] and aging [16], as the subjects were older at the time of death.

The occurrence of VFs was more frequent in T12 and L1 regions, representing the transition zone between the thoracic and lumbar spine. The thoracolumbar junction is the site of stress concentration where the transition from thoracic kyphosis to lumbar lordosis occurs [17]. The VFs were most frequently found at the thoracolumbar junction in previous studies [18, 19]. The VFs of the middle thoracic spine were observed with greater frequency within the thoracic spine. Moreover, the VFs at the thoracolumbar junction can alter the entire spinal column alignment, which redistributes the loads across the vertebrae, leading to increased loading on the middle thoracic vertebrae, which may induce secondary thoracic VFs [20, 21].

In the current study, a history of surgical intervention for femoral neck fractures was found in 7.5% and 21.5% of men and women, respectively. The prevalence of osteoporosis at the femoral neck was 11.1%–13.5% and 49.0%–51.5% for men and women aged ≥ 80 years, respectively, in the baseline survey (2005–2006) and fourth survey (2015–2016) [22]. Reduced bone density is linked to an increased risk of hip fractures [23]. Hip fractures are more prevalent in individuals in their 80 s and 90 s, with documented annual incidence rates [24–26]. Nevertheless, the prevalence of this condition at death in the Japanese population has yet to be investigated. In this study, the prevalence of subjects with a history of surgical intervention for femoral neck fractures, while conservative medical management could not be determined, provides an approximate lifetime prevalence of femoral neck fractures, which are treated surgically in 94% of cases [27]. Although hip fractures are more prevalent in western Japan, where residents have an inadequate intake of milk and natto, the fermented soybean (traditional Japanese food), containing high vitamin K, the prevalence of hip fractures in Shimane Prefecture, in western Japan, where the study subjects resided was similar to the national average [28]. VFs have been linked to an increased risk of subsequent hip fractures [29, 30]. We also showed an association between a history of surgical intervention for hip fractures and the number and severity of VFs.

Notably, this study has limitations. First, the general Japanese population may not be adequately represented in the present study as our subjects had previously joined the charitable association of cadaver donation to our medical school for anatomy dissection during their lifetime. The place of death occurs in facilities, which may reflect the current situation of a growing number of single elderly people in Japan and the increasing number of members in the charitable association of cadaver donations from this population. However, the mean age at death was almost identical to the mean Japanese life expectancy in 2019, which was 81.4 years for men and 87.5 years for women. Furthermore, up to the end of 2019, when this study was conducted, preceded the onset of the COVID-19 pandemic and the subsequent implementation of restrictions on the acceptance of cadavers with pneumonia as the direct cause of death. Cadavers were accepted for study, except in special cases, such as road accidents.

Second, Shimane Prefecture, the place of residence of the participants, is in western rural Japan and experiences shorter daylight hours than other regions of Japan. Differences in physical activity intensity and food intake between this region and others in Japan may have contributed to the observed prevalence of VFs and hip fractures.

In conclusion, our findings showed the VF prevalence at death through AiCT of cadavers donated for anatomical dissection. The prevalence was higher than that reported in previous studies of the same age group. Women had a higher prevalence of severe VFs. Moreover, the most frequent VFs were those at T12 and L1, the thoracolumbar junction, whereas those at T3–L5 were more prevalent in women. The presence of VFs and Grade 3 fractures was associated with the administration of surgical treatment for femoral neck fractures.
